# Impact of cytokeratin-20 and carcinoembryonic antigen mRNA detection by RT-PCR in regional lymph nodes of patients with colorectal cancer

**DOI:** 10.1054/bjoc.2000.1442

**Published:** 2000-10-26

**Authors:** R Rosenberg, A Hoos, J Mueller, H Nekarda

**Affiliations:** Department of Surgery, Klinikum Rechts der Isar of the Technical University, Munich, Germany

**Keywords:** colorectal cancer, CEA, CK-20, RT-PCR, tumour cell involvement, prognosis

## Abstract

The reported rates for tumour cell involvement in the locoregional lymph nodes of colorectal cancer vary greatly, depending on the method used and case selection. In order to further evaluate the clinical value of molecular biologic detection of tumour cells we investigated 102 histologically tumour-free (pN0) regional lymph nodes from 51 consecutive, completely resected (UICC R0) colorectal carcinoma specimens for the presence of tumour cell mRNA by RT-PCR specific for carcinoembryonic antigen (CEA) and cytokeratin 20 (CK-20). Two lymph nodes located nearest to the primary tumour were investigated in each case. CK-20 mRNA was found in 31 of 51 patients (60.8%) and CEA mRNA in 30 of 51 patients (58.8%), respectively. Identical transcription patterns of CK-20 and CEA mRNA (both positive or both negative) were found in 38 of 51 patients (74.5%). There was a significantly higher proportion of cases with CEA positivity in the lymph nodes of tubulopapillary than of mucinous adenocarcinomas (*P*< 0.03). Detection of CK-20 and CEA mRNA correlated in nine of 12 cases (75.0%) with the risk of tumour recurrence (not significant) and showed a tendency towards shorter disease-free survival by univariate analysis (not significant). Our data indicate that CK-20 and CEA mRNA detection by RT-PCR may prove useful for the prediction of tumour recurrence of patients with pN0 colorectal carcinoma, although neither reach statistical significance in this series of patients. © 2000 Cancer Research Campaign


*British Journal of Cancer* (2000) **83**(10), 1323–1329
© 2000 Cancer Research Campaign
doi: 10.1054/ bjoc.2000.1442, available online at http://www.idealibrary.com on IDEAL®

### Impact of cytokeratin-20 and carcinoembryonic antigen mRNA detection by RT-PCR in regional lymph nodes of patients with colorectal cancer

**R Rosenberg, A Hoos, J Mueller and H Nekarda**

Department of Surgery, Klinikum Rechts der Isar of the Technical University (Chairman: JR Siewert), Munich, Germany

**Summary** The reported rates for tumour cell involvement in the locoregional lymph nodes of colorectal cancer vary greatly, depending on the method used and case selection. In order to further evaluate the clinical value of molecular biologic detection of tumour cells we investigated 102 histologically tumour-free (pN0) regional lymph nodes from 51 consecutive, completely resected (UICC R0) colorectal carcinoma specimens for the presence of tumour cell mRNA by RT-PCR specific for carcinoembryonic antigen (CEA) and cytokeratin 20 (CK-20). Two lymph nodes located nearest to the primary tumour were investigated in each case. CK-20 mRNA was found in 31 of 51 patients (60.8%) and CEA mRNA in 30 of 51 patients (58.8%), respectively. Identical transcription patterns of CK-20 and CEA mRNA (both positive or both negative) were found in 38 of 51 patients (74.5%). There was a significantly higher proportion of cases with CEA positivity in the lymph nodes of tubulopapillary than of mucinous adenocarcinomas ($P < 0.03$). Detection of CK-20 and CEA mRNA correlated in nine of 12 cases (75.0%) with the risk of tumour recurrence (not significant) and showed a tendency towards shorter disease-free survival by univariate analysis (not significant). Our data indicate that CK-20 and CEA mRNA detection by RT-PCR may prove useful for the prediction of tumour recurrence of patients with pN0 colorectal carcinoma, although neither reach statistical significance in this series of patients. © 2000 Cancer Research Campaign

**Keywords:** colorectal cancer; CEA; CK-20; RT-PCR; tumour cell involvement; prognosis

Colorectal cancer continues to increase in incidence and the disease remains the second most common cause of cancer-related deaths (Steele, 1994). The prognosis of patients with colorectal cancer depends on the TNM stage of the tumour, in particular lymph-node involvement, and whether or not the tumour is completely resected (R0) (Ratto et al, 1998). Despite careful histopathological assessment of resected lymph nodes, up to 20% of all node-negative patients with a curative resection develop tumour recurrence (Hermanek, 1995). The best explanation for this is that tumour cells have already spread beyond the area of resection before removal of the tumour, but are undetectable by routine staging methods. Efforts to detect such minimal residual disease have centered on the use of highly sensitive techniques for tumour cell detection in lymph nodes, bone marrow and peripheral blood.

Various methods including fat clearance (Cawthorn et al, 1986), multiple serial sections (Zhang et al, 1998) and immunohistochemistry (Davidson et al, 1990; Cutait et al, 1991; Calaluce et al, 1998; Natsugoe et al, 1998; Oeberg et al, 1998), have been developed, but none of these have become useful in a daily routine examination of lymph nodes.

RT-PCR (reverse transcriptase polymerase chain reaction) for the detection of mRNA has been proven to be an effective and highly sensitive method for detecting tumour-associated mRNA transcription (Raj et al, 1998). Despite various clinical studies using RT-PCR to detect 'micrometastatic tumour cells' in lymph nodes, the clinical significance of these results is still unclear (Mori et al, 1995; 1998; Demeure et al, 1998; Futamura et al, 1998; Liefers et al, 1998). One reason for this may be that the high sensitivity of the method allows the detection of mRNA, but the tumour cells which express this mRNA may not be responsible for the development of recurrent disease. It is possible that some detected tumour cells are biologically inactive, due to an intrinsic dormancy or an immunological filter function of lymph nodes. In addition, false-positive detection may result from amplification of non-cancer-specific tissue (illegitimate transcription) (Henke and Loening, 1998). Therefore further evaluation of the clinical and prognostic value of mRNA detection by RT-PCR is needed.

One of the most commonly used targets of RT-PCR for tumour cell detection is the carcinoembryonic antigen (CEA) (Raj et al, 1998). But few studies using CEA RT-PCR for detection of tumour cell mRNA in lymph nodes have been performed in colorectal carcinoma (Mori et al, 1995; 1998; Futamura et al, 1998; Liefers et al, 1998). A valuable clinical correlation between CEA mRNA detection and prognosis has only been described in one study with a small cohort of patients (Liefers et al, 1998).

Cytokeratins (CK) are also a frequent target for RT-PCR tumour cell detection, but detection of CK is complicated by the existence of CK-18 and 19 pseudogenes (Neumaier et al, 1995; Ruud et al, 1999). CK-20 has been shown to be a specific marker of gastrointestinal epithelial cells (Moll et al, 1992), has been used for the detection of tumour cell involvement (Futamura et al, 1998; Funaki et al, 1998) and has the advantage that no CK-20 pseudogenes have been identified. Recently, Jung et al (1999) described a background mRNA

*Received 24 January 2000*
*Revised 6 July 2000*
*Accepted 11 July 2000*

*Correspondence to:* H Nekarda, Department of Surgery, Klinikum Rechts der Isar, Ismaringerstr. 22, 81675 Munich, Germany expression of CK-20 in granulocytes of non-cancer patients, which has to be further evaluated. Combination of various mRNA targets may also help to improve the clinical usefulness of the analysis.

We carried out the present study in order to further investigate the clinical value of CEA and CK-20 mRNA detection by RT-PCR in regional lymph nodes of patients who had undergone a complete resection of their colorectal tumour and had no detectable lymph-node metastasis by routine methods. We limited our analysis to two peritumoural lymph nodes per case to simulate a technique which might be applicable to a routine clinical setting in the future. The results of mRNA detection were compared with tumour recurrence and patient survival data.

### MATERIAL AND METHODS

### Patient group

The patient study group consisted of 51 consecutive patients with colorectal cancer who underwent resection in our department between 1990–1992 (Table 1). The mean of patient age was 60.4 years and there were 25 males and 26 females. Tumour recurrence and survival data were available from all 51 patients with a median follow-up time of 94 months (range 10–102 months). The overall 5-year survival rate for the 51 patients was 87.5%. Twelve patients had developed recurrent disease during the follow-up period. Distant metastases were confirmed in nine patients (liver metastases in eight cases and lung metastasis in one case), local recurrence with or without lymph-node metastasis, was detected in three patients (Table 1). Of these 12 patients none were alive at the end of follow-up, 11 patients (91.7%) had died of recurrent disease and one patient died of a cause unrelated to his cancer. The control group consisted of 10 patients with chronic inflammatory bowel disease who were operated upon in our department.

### Tumours

In order to be included in the study, the tumours needed to be entirely resected (UICC R0) and the lymph nodes found to be free of tumour cells (pN0) by histopathological examination. Thirty-eight of the 51 tumours were located in the colon and 13 were from the rectum. The resection procedure performed consisted of one abdominoperineal resection, 12 (low) anterior resections, seven sigmoid resections, 11 left hemicolectomies and 20 right (radical) hemicolectomies. The extent of the lymph-node dissection was performed according to oncological recommendations (Ruo et al, 1998). According to staging based on the TNM classification, 15 tumours (29.4%) were stage I (pT1 = 5, pT2 = 10) and 36 (70.6%) were stage II (pT3 = 32, pT4 = 4). Histologically, 43 (84.3%) tumours were tubulopapillary adenocarcinomas, the other eight (15.7%) were mucinous adenocarcinomas. Between six and 72 lymph nodes were resected along with the tumours (median of 29), seven of the specimens (13.7%), had less than 12 removed nodes. Immunohistochemical CEA and CK-20 staining was performed on all 51 primary tumours, which confirmed that all of the primary colorectal carcinomas expressed both CEA and CK 20.

### Lymph-node sampling

To simulate a technique that might be clinically feasible two lymph nodes from each resection specimen (a total of 102 lymph nodes) were taken, that were located nearest to the primary tumour (peritumoural lymph nodes). After division into two halves, one half was used for routine pathological examination and the other was immediately frozen and stored in liquid nitrogen until use. Thirty cryostat sections of 15 μm thickness were prepared for RNA extraction from each frozen tissue sample. One 6 μm frozen section was prepared before and one after the 30 serial sections and examined with a haematoxylin and eosin (HE) staining to confirm that no tumour cells were present histologically.

### RNA extraction

The silica gel-based membrane system RNeasy (Quiagen) was used for extraction of total RNA from the lymph-node samples and the tumour cell lines. The amount of total RNA was measured with a spectrophotometer at 260 nm and 280 nm wavelengths. Approximately 40–60 μg of total RNA from each lymph-node sample was extracted.

### Controls

Tumour cell lines were used as positive controls. The CEA- and CK-20-positive tumour cell lines CaCo-2 (colorectal cancer) and MCF-7 (breast cancer) were grown in cell culture in RPMI or DMEM medium, and were counted in a haematocytometer (Neubauer chamber) prior to RNA extraction. The expression level of CEA in the supernatant of the MCF-7 cells was approximately double of that of the CaCo-2 cells. The pooled RNA of 14 primary colorectal carcinomas displaying high levels of CEA and CK-20 was used as an additional positive control. As negative controls, mRNA from lymph nodes obtained from patients with chronic inflammatory bowel disease were examined to evaluate the level of CEA expression in normal lymph nodes of non-cancer patients. These lymph nodes remained negative for both markers throughout the experiments. Efficient amplification of the RNA was monitored by a control PCR for β2-microglobulin using the primers M1: 5′-CCT GAA TTG CTA TGT GTC TGG GTT TCA-3′ and M2: 5′-GGA GCA ACC TGC TCA GAT ACA TCA AAC-3′. Efficient RNA amplification was consistently obtained from all samples.

### Primers

CEA oligonucleotide primers for RT-PCR based on the published sequence of the CEA gene (Schrewe et al, 1990) were used for CEA mRNA detection as published by Gerhard et al (1994). For detection of CK-20 mRNA, a set of published CK-20 specific primers was used (Funaki et al, 1998).

### RT-PCR reactions

The RT-PCR reactions were performed in a Landgraf Varius-V thermocycler with the Titan™ One Tube RT-PCR and PCR Master kits (Boehringer Mannheim, Germany). The reactions were carried out in a reaction volume of 50 μl with 2 μg of total RNA per sample. For CEA amplification the following conditions were used: reverse transcription at 50°C for 30 min, template denaturation at 94°C for 2 min, 10 cycles including denaturation at 94°C for 30 s, annealing at 69°C for 30 s and elongation at 68°C for 45 s, another 20 cycles under the same conditions followed by

 © 2000 Cancer Research Campaign

**Table 1** Patient characteristics in comparison to tumour mRNA detected by RT-PCR

| Patients | UICC stage | Histology | Resected nodes | pT | Operation | RT-PCR detection | | Outcome | | Disease-free survival (months) |
|---|---|---|---|---|---|---|---|---|---|---|
| | | | | | | CEA | CK-20 | Vital | Disease status | |
| 01 | II | T | 31 | 4 | 1 | + | + | DOD | recurrence[a] | 39 |
| 02 | II | T | 57 | 3 | 4 | + | + | alive | NED | 101 |
| 03 | II | M | 52 | 3 | 5 | – | + | alive | NED | 128 |
| 04 | I | T | 32 | 2 | 3 | + | + | DOD | recurrence[b] | 24 |
| 05[a] | I | T | II | 1 | 2 | + | – | DOD | recurrence[b] | 71 |
| 06 | II | M | 40 | 3 | 5 | + | + | DOD | recurrence[b] | 56 |
| 07 | II | M | 52 | 3 | 4 | – | – | alive | NED | 122 |
| 08 | II | T | 12 | 4 | 2 | + | + | alive | NED | 122 |
| 09 | II | T | 32 | 3 | 2 | – | – | alive | NED | 121 |
| 10 | II | T | 44 | 3 | 5 | – | – | alive | NED | 60 |
| 11 | II | T | 17 | 3 | 2 | + | + | alive | NED | 119 |
| 12 | II | T | 72 | 4 | 4 | + | – | alive | NED | 119 |
| 13 | II | T | 22 | 3 | 3 | + | + | alive | NED | 119 |
| 14 | II | T | 32 | 3 | 3 | – | – | DUC | NED | 22 |
| 15 | II | T | 61 | 3 | 5 | – | + | alive | NED | 116 |
| 16 | I | T | 43 | 2 | 5 | + | + | DUC | recurrence[b] | 29 |
| 17 | II | T | 14 | 3 | 4 | – | – | alive | NED | 115 |
| 18 | II | T | 21 | 3 | 5 | + | + | DUC | NED | 44 |
| 19 | I | T | 19 | 1 | 5 | – | + | alive | NED | 42 |
| 20[a] | II | T | 7 | 3 | 4 | + | + | alive | NED | 103 |
| 21 | II | T | 33 | 3 | 4 | – | – | DUC | NED | 10 |
| 22 | II | T | 24 | 3 | 2 | + | + | DOD | recurrence[c] | 16 |
| 23 | II | T | 44 | 3 | 4 | + | – | alive | NED | 102 |
| 24 | II | T | 29 | 3 | 5 | – | – | alive | NED | 102 |
| 25 | II | T | 35 | 3 | 2 | + | + | DOD | recurrence[b] | 61 |
| 26 | I | T | 31 | 2 | 4 | + | + | alive | NED | 100 |
| 27 | II | M | 22 | 3 | 4 | – | – | DOD | recurrence[c] | 16 |
| 28 | I | M | 39 | 2 | 2 | – | + | DOD | recurrence[b] | 66 |
| 29 | II | T | 25 | 3 | 5 | + | + | alive | NED | 96 |
| 30 | II | T | 34 | 3 | 5 | + | + | alive | NED | 96 |
| 31 | I | T | 49 | 1 | 4 | + | – | alive | NED | 95 |
| 32 | II | M | 30 | 3 | 5 | – | + | alive | NED | 95 |
| 33 | II | T | 23 | 3 | 5 | – | – | alive | NED | 94 |
| 34 | II | T | 28 | 3 | 5 | – | – | DUC | NED | 54 |
| 35 | I | T | 33 | 2 | 2 | + | + | DUC | NED | 75 |
| 36 | II | T | 26 | 4 | 3 | + | – | alive | NED | 94 |
| 37[a] | II | T | 8 | 3 | 3 | + | + | DOD | recurrence[b] | 40 |
| 38 | II | M | 38 | 3 | 5 | + | + | alive | NED | 92 |
| 39 | I | T | 14 | 2 | 2 | + | + | alive | NED | 91 |
| 40 | II | T | 25 | 3 | 5 | – | – | alive | NED | 91 |
| 41 | II | T | 38 | 3 | 3 | + | – | alive | NED | 89 |
| 42[a] | I | T | 7 | 2 | 2 | – | – | alive | NED | 88 |
| 43 | I | T | 18 | 2 | 5 | – | + | alive | NED | 88 |
| 44 | II | T | 51 | 3 | 5 | + | + | alive | NED | 87 |
| 45[a] | I | T | 11 | 1 | 2 | + | + | DOD | recurrence[c] | 18 |
| 46 | I | T | 20 | 2 | 3 | + | + | alive | NED | 86 |
| 47[a] | II | M | 10 | 3 | 5 | – | + | alive | NED | 86 |
| 48 | II | T | 29 | 3 | 4 | + | + | alive | NED | 85 |
| 49 | II | T | 20 | 3 | 2 | + | + | alive | NED | 84 |
| 50 | I | T | 50 | 1 | 5 | – | – | alive | NED | 85 |
| 51[a] | I | T | 6 | 2 | 5 | – | – | DOD | recurrence[b] | 2 |

[a] = less than 12 resected lymph nodes; T = tubulo-papillary; M = mucinous adenocarcinoma; Operation: 1 = abdominoperineal resection; 2 = (low) anterior resection; 3 = sigmoid resection; 4 = left (radical) hemicolectomy; 5 = right (radical) hemicolectomy. Recurrent site: [b]distant metastasis (liver $n$ = 8, lung $n$ = 1), [c]local recurrence; DOD = died of disease; DUC = died of cancer-unrelated cause; NED = no evidence of disease

5 min of prolonged elongation at 68°C. The reaction conditions for the CK-20 RT-PCR were as follows: reverse transcription at 50°C for 30 min, template denaturation for 5 min at 93°C, 10 cycles of denaturation (1 s at 94°C), annealing (20 s at 63°C), elongation (10 s at 72°C), 35 more cycles under the same conditions and a final step for 5 min at 72°C.

Gel electrophoresis was performed on 2% agarose gels. As length standards HPA II, Dra I and Hind III digested DNA of pUCBM21 (DNA-length standard VIII, Boehringer Mannheim) were used. The amplified DNA fragments were 160 bp for the CEA primer pairs and 370 bp for the CK-20 primer pairs. Sequencing of these PCR products confirmed that the selected portion of the target mRNA had been amplified (data not shown). During initial testing of the method, nested PCR with additional primers (not shown) was performed, but did not improve the results, so the analysis of the lymph nodes was performed with RT-PCR alone.

© 2000 Cancer Research Campaign

### Sensitivity testing

Using the colon and breast cancer cell lines CaCo-2 and MCF-7, dilution series of RNA equivalent to $10^6$, $10^5$, $10^4$, $10^3$, $10^2$, 10 and one tumour cells in $10^6$ mononuclear cells were performed.

### Statistical analysis

For group comparisons the $\chi^2$-test was used. A difference of $P < 0.05$ was considered to be significant. Group oriented curves for recurrence-free survival were calculated according to the method of Kaplan and Meier. The significance of differences between groups was examined using the log-rank test. Survival analysis was performed for the total patient group ($n = 51$) and, in addition, for the subgroup of 44 patients, who had at least 12 resected lymph nodes, according to the recommendations of the UICC.

### RESULTS

### Sensitivity testing

Using the cell lines CaCo-2 and MCF-7, the dilution series of RNA indicated that both primer pairs were able to detect an amount of mRNA equivalent to one tumour cell in $10^6$ mononuclear cells (Figure 1).

### Detection

One hundred and two lymph nodes of 51 patients with colorectal cancer and 20 lymph nodes of 10 patients with chronic inflammatory bowel disease were analysed. All lymph nodes from the cases with inflammatory, non-neoplastic disease were negative for both CEA- and CK-20 mRNA.

The examination of the lymph nodes of 51 patients with colorectal disease detected CK-20 mRNA in 31 patients (60.8%) (Table 2, Figure 2). CEA mRNA was positive in 30 cases by RT-PCR analysis (58.8%). A total of 37 patients (72.5%) expressed either CEA or CK-20, or both. Identical expression patterns for CK-20 and CEA (both negative or both positive) were found in 38 of 51 patients (74.5%), with the remaining 13 cases (25.5%) showing discrepant results between the two sets of primers.

### Correlation with histopathologic findings

A comparison of CEA RT-PCR positivity in lymph nodes and the histologic type of the tumour showed that a significantly higher percentage of regional lymph nodes of tubulopapillary adenocarcinoma (28 of 43 = 65.1%) vs mucinous adenocarcinomas (2 of 8 = 25%) expressed CEA ($P \leq 0.03$). No such significant difference according to histologic type was seen for CK-20. Also, no correlation was found between positive RT-PCR reactions and tumour site, depth of invasion (pT category), CEA serum level, presence of lymphatic vessel invasion or the histologic grade of the tumour.

### Correlation with recurrence and prognosis

Twelve of 51 patients (23.5%) developed recurrent disease (Table 1) and died during the follow-up period (median follow-up time 94 months). Of these 12 patients with recurrent disease, nine had positive lymph nodes for CK-20 mRNA and CEA mRNA (75.0%). Three cases were not detected by the CK-20 and CEA primers (25.0%), a difference which was statistically not significant. The site of recurrence (nine cases with distant metastases and three with local recurrent disease) had no impact on the detection rates (Table 1). Eight of the nine patients (89%), who developed distant metastasis were detected either by CK-20 PCR or by CEA PCR. Of the three patients who developed local recurrent disease, two patients (67%) were detected by RT-PCR analysis. Positive mRNA detection in cases without tumour recurrence during the follow-up period ($n = 39$) was found in 22 cases (56.4%) for CK-20 and in 21 cases (53.8%) for CEA.

In order to avoid the problem of stage migration (Will Rodgers phenomenon, Schlag, 1998), the results of the RT-PCR reactions of seven patients (13.7%) with fewer than 12 resected lymph nodes (pNx) were excluded and also analysed for clinical outcome. Eight of 44 remaining patients (18.2%) had developed recurrent disease. Of these eight patients, seven patients were identified to have positive lymph nodes for CK-20 mRNA (87.5%). Only one case (12.5%) was not detected by the CK-20 primer ($P = 0.09$). For CEA, six of the eight patients with recurrent disease had positive lymph nodes by RT-PCR (75.0%) and two cases were not detected by CEA RT-PCR (25.0%).

No statistical difference concerning the rate of positivity between patients with and without recurrence was seen for CEA

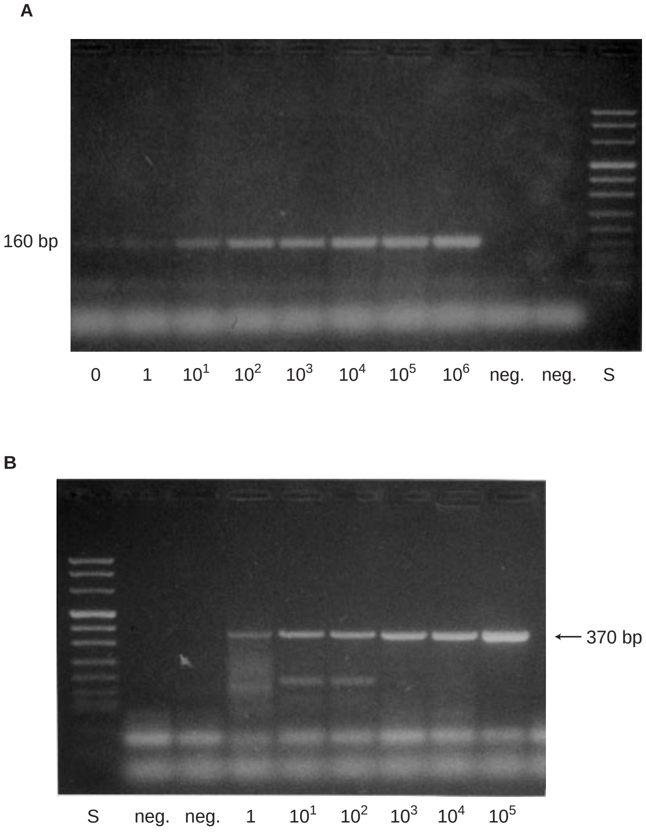

**Figure 1** 10-fold serial dilutions of RNA equivalent to $10^5$–0 tumour cells (cell line MCF-7) in $10^6$ mononuclear cells which demonstrates the sensitivity of the RT-PCR assays for CEA (**A**) and CK-20 (**B**). Both methods were able to detect one tumour cell in $10^6$ normal cells. DNA fragments were run in a 2% agarose gel. Negative controls obtained from lymph nodes with chronic inflammatory bowel disease were shown (marked as neg.). The DNA length standard VIII (Boehringer Mannheim) is shown in lane S

© 2000 Cancer Research Campaign

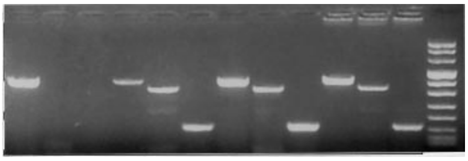

**Figure 2** Amplified DNA fragments from the lymph-node samples of two patient (patient 1 and 16, Table 1) obtained by the two RT-PCR methods (lanes 7–12). Negative control of patients with inflammatory bowel disease is demonstrated on lanes 1–3. As positive control pooled RNA of colorectal carcinoma was used, shown on lanes 4–6. The CEA-specific cDNA fragments amplified by RT-PCR had a length of 160 bp (lanes 6, 9, 12), the CK-20 specific fragment was 370 bp long (lanes 5, 8, 11). In lanes 1, 4, 7 and 10 the β2-microglobulin-specific fragment is seen, indicating efficient RNA amplification. The DNA length standard VIII is shown in lane 13

and CK-20. The combination of the results for CK-20 and CEA resulted in no advantage as well. A longer disease-free survival was seen for patients with lymph nodes negative for CK-20 RT-PCR vs patients with lymph nodes positive by CK-20 RT-PCR reaction (n.s.). CEA RT-PCR showed also a tendency to identify patients with longer disease-free survival (Figure 3).

### DISCUSSION

Based on our results and data from the literature, RT-PCR for the detection of CK-20 mRNA and CEA mRNA in clinically sampled regional lymph nodes of pN0 colorectal cancer appears to be a promising tool for the identification of patients at high risk of tumour recurrence. Our study included 51 cases of colorectal cancer with a long follow-up period of 94 months, in which the lymph nodes were found to be negative for tumour by routine histopathologic methods. We limited our analysis to two lymph nodes per case, which were selected as the nearest detectable nodes to the primary tumour. We suggest that such a strategy might be applicable to a routine clinical setting. Our results support the clinical usefulness of such a strategy, because the RT-PCR examination of only two resected peritumoural lymph nodes predicts the risk of tumour recurrence in 75% of cases.

The significance of 'micrometastatic' detection by RT-PCR as a marker for prediction of distant metastasis or local recurrence is still unclear. Our results show a tendency regarding distant metastasis. CEA was used as a target for detection of tumour mRNA because of existing clinical data. CK-20 was chosen as an additional target since, unlike CK-18 and CK-19, there are no pseudogenes and it is almost always expressed by colorectal carcinomas (Moll et al, 1992). The potential role of CK-20-specific RT-PCR to evaluate 'micrometastasis' was demonstrated by Yun et al (2000), who validated CK-20 as a specific marker of submicroscopic lymph-node metastases in colorectal cancer by its correlation to K-RAS mutations. This underlined the concept that what is detected by RT-PCR represents occult metastatic disease.

Previous studies have described the use of RT-PCR targeting cytokeratins or CEA for the detection of occult tumour cells, but a lack of clinical data or the various methods which have been used have complicated the assessment of the clinical significance of these findings (Table 3). Mori et al (1995; 1998) reported the

**Table 2** Detection of CK-20 mRNA and CEA mRNA in lymph nodes of patients with and without recurrent disease during follow-up (not significant)

| RT-PCR | | | No recurrence *n* = 39 (76.5%) | | Recurrence *n* = 12 (23.5%) | |
|---|---|---|---|---|---|---|
| | CK-20 | CEA | CK-20 | CEA | CK-20 | CEA |
| Neg. | 20/51 (39.2%) | 21/51 (41.2%) | 17/39 (43.6%) | 18/39 (46.2%) | 3/12 (25.0%) | 3/12 (25.0%) |
| Pos. | 31/51 (60.8%) | 30/51 (58.8%) | 22/39 (56.4%) | 21/39 (53.8%) | 9/12 (75.0%) | 9/12 (75.0%) |

detection of tumour cells in the regional lymph nodes of gastrointestinal and breast carcinomas using the same primers for CEA RT-PCR which we used in our study (Gerhard et al 1994). The authors concluded that RT-PCR was a powerful tool for the detection of tumour cell deposits in lymph nodes, but only a few of the tumours examined were colorectal carcinomas and, additionally, no reliable statement about the clinical impact of tumour cell detection by this method could be made (Mori et al, 1995; 1998). A combined RT-PCR analysis for CEA and CK-20 mRNA in lymph nodes from 13 patients with colorectal cancer in another study revealed that 100% of these had positive lymph nodes by one or both primers (Table 3), but no survival analysis was performed (Futamura et al, 1998). The only study which demonstrated a significant correlation between the presence of CEA mRNA in regional lymph nodes and survival was performed by Liefers et al (1998). This study included only 26 patients with stage II colorectal cancer and did not provide a practical approach for routine clinical use.

Our RT-PCR results for CEA, utilizing primers which have been used in above-mentioned previous studies (Gerhard et al, 1994), showed different expression patterns in 25% of our samples in comparison to that detected with CK-20 RT-PCR. We detected CK-20 and/or CEA mRNA in the lymph nodes of 37 of 51 patients (72.5%), while 31 and 30 cases, respectively, were positive for either one of the markers. Expression patterns between CEA and CK-20 (both negative or both positive) were identical in 38 of 51 cases (74.5%). The high RT-PCR detection of CK-20 and/or CEA mRNA of 75% in lymph nodes of patients who developed recurrent disease or distant metastasis during the long follow-up time, indicate a potential role for CK-20 and CEA as markers for detection of prognostically relevant tumour cell deposits in regional lymph nodes of colorectal cancer patients. In addition, our CEA data reflect the complexity of CEA expression and its potential heterogeneity in tumour-associated lymph nodes. They also show differences in expression between histological subtypes of tumours (tubulopapillary and mucinous adenocarcinomas).

Although studies which have shown a significant correlation of RT-PCR tumour mRNA detection and prognosis are reported in the literature, the RT-PCR method still confronts us with problems that need to be solved. One is that detection of tumour mRNA may not always show a correlation with patient survival or the probability of tumour recurrence. In our series the high rate of detection of CEA or CK-20 mRNA in 53% or 56% of patients who developed no recurrent disease suggests either that the detected tumour cells were not of clinical significance because of their biological characteristics or there was contamination of the specimen during the processing of the lymph nodes ('false-positive' results). Thus, mRNA detection did not reach statistical significance for either the prediction of tumour recurrence or disease-free survival.

© 2000 Cancer Research Campaign 

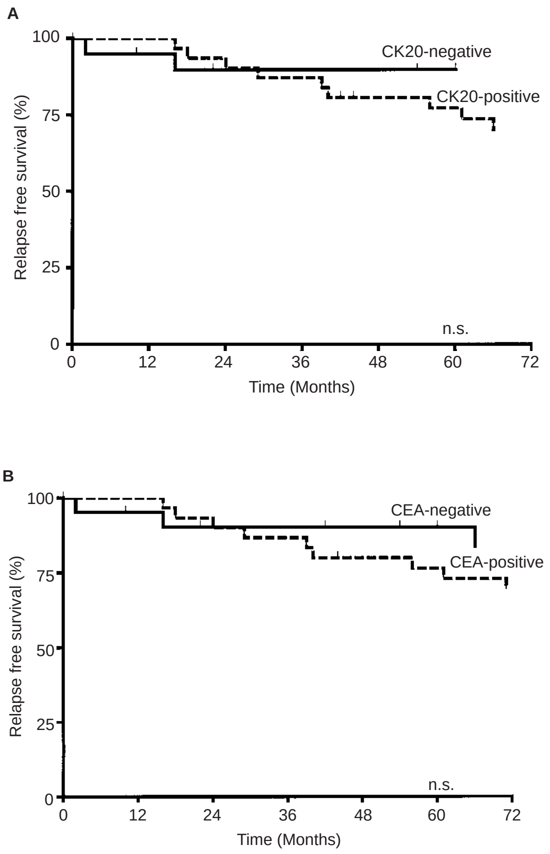

**Figure 3** Kaplan–Meier curve showing disease-free survival of patients with lymph nodes positive and negative for CK-20 mRNA (**A**) and CEA mRNA (**B**). The patients with a negative CK-20 or CEA RT-PCR have a tendency for longer disease-free survival than those with CK-20 or CEA positivity (n.s.) (CK 20-negative relapse rate 3/20 (15%), CK20-positive relapse rate 9/31 (29%), CEA-negative relapse rate 3/21 (14%), CEA-positive relapse rate 9/30 (30%))

These results fit with the concept that detected tumour cells in the lymph nodes are not biologically active metastases, but are ‘epiphenomena’, with very little impact on patient prognosis. Based on the morphology of immunohistochemically detected tumour cell deposits, these can be classified as either micrometastases, when they display a surrounding reaction and are presumably biologically active, or as tumour cell microinvolvement, when such a reaction is not present and the cells may be in a biologically inactive state (Natsugoe et al, 1998). This morphologic distinction between types of involvement is not possible with RT-PCR based methods.

A further problem for sensitive molecular techniques may be the possibility that some normal cells express very small amounts of target mRNA which may interfere with specific expression by epithelial or tumour cells (illegitimate transcription). This can result in false-positive results by RT-PCR (Jung et al, 1999). However, based on the fact that our negative control (lymph nodes of patients with chronic inflammatory bowel disease) was consistently negative, we did not observe this phenomenon. A solution for the problem of illegitimate transcription may be the use of quantitative PCR methods by which a cut-off level for a given primer pair could be established in order to consider a given RT-PCR reaction to be positive.

The high rate of mRNA detection in lymph nodes of patients without recurrence, which is also observed in studies reported in the literature (Futamura et al, 1998), could possibly be explained through postoperative contamination, as described. In comparison to immunohistochemical analysis, molecular biological methods do not allow morphological evaluation of lymph nodes and even haematoxylin-eosin staining, as performed in our study, cannot exclude single-cell contamination. This phenomenon can influence the results of a RT-PCR assay (Fujiwara et al, 2000).

In summary, this study confirms that the examination of tumour-associated lymph nodes in colorectal cancer by RT-PCR can be clinically relevant since it can predict tumour recurrence. If this procedure is only performed on a limited number of lymph nodes, it could prove useful as a potential strategy for the routine clinical setting. The selection of the target gene for mRNA detection is an important factor, which has to be further evaluated in future studies, since CK-20 and CEA showed little differences for the prediction of tumour recurrence. Studies with larger patient groups will be needed to confirm our results and to demonstrate statistical significance for the prognostic significance of these markers. In addition, immunohistochemical staining of lymph-node sections parallel with PCR analysis is needed for morphological examination of sections, allowing the role of potential contamination of specimens with tumour cells to be demonstrated. Finally, the establishment of quantitative methods for the definition of biologically relevant tumour deposits in the lymph nodes of colorectal carcinoma might be an interesting addition to this promising technique.

### ACKNOWLEDGEMENTS

Dedicated to our department chief Professor Dr JR Siewert on the occasion of his 60th birthday. We thank Mrs Seidl and Mrs Poehlmann for their expert technical assistance.

**Table 3** Study comparison showing the available data on clinical significance of RT-PCR detection of CK-20 or CEA mRNA in locoregional lymph nodes of colorectal cancer (pN0)

| Study | Patients (*n*) | Lymph nodes (*n*) | RT-PCR positive LN for CK-20 *n* | CK-20 % | CEA *n* | CEA % | Patients with PCR pos. cells *n* | % | Impact on prognosis |
|---|---|---|---|---|---|---|---|---|---|
| Futamura 1998 | 13 | 202 | 81 | 40.0 | 95 | 47.0 | 13 | 100.0 | n.e. |
| Liefers 1998 | 26 | 192 | n.e. | – | 36 | 18.7 | 14 | 53.8 | yes |
| Mori 1998 | 65[a] | 406 | n.e. | – | 245 | 60.3 | 44 | 67.6 | yes |
| Own results | 51 | 102 | 62 | 60.8 | 60 | 58.8 | 37 | 72.5 | n.s. |

[a]Colorectal (*n* = 20), gastric (*n* = 31), breast (*n* = 10) and oesophageal (*n* = 4) cancer patients; n.e. = not examined; n.s. = not significant

 *© 2000 Cancer Research Campaign*

### REFERENCES

Calalucc R, Miedema BW and Yesus YW (1998) Micrometastases in colorectal carcinoma: a review. *J Surg Oncol* **67**: 194–202

Cawthorn SJ, Gibbs NM and Marks CG (1986) Clearance technique for the detection of lymph nodes in colorectal cancer. *Br J Surg* **73**: 58–60

Cutait R, Alves VAF and Lopes LC (1991) Restaging of colorectal cancer based on the identification of lymph node micrometastases through immunperoxidase staining of CEA and cytokeratins. *Dis Colon Rectum* **34**: 917–920

Davidson BR, Sams VR and Styles J (1990) Detection of occult nodal metastases in patients with colorectal carcinoma. *Cancer* **65**: 967–970

Demeure MJ, Doffek KM, Komorowski RA and Wilson SD (1998) Adenocarcinoma of the pancreas. Detection of occult metastases in regional lymph nodes by a polymerase chain reaction-based assay. *Cancer* **83**: 1328–1334

Fujiwara Y, Ookka M, Sugita Y, Sakita I, Tamaki Y and Monden M (2000) Prevention of cross-contamination during sampling procedure in molecular detection for cancer micrometastasis. *Cancer Lett* **153**: 109–111

Funaki NO, Tanaka J, Ohshio G, Onodera H, Maetani S and Imamura M (1998) Cytokeratin 20 mRNA in peripheral venous blood of colorectal carcinoma patients. *Br J Cancer* **77**: 1327–1332

Futamura M, Takagi Y, Koumura H, Kida H, Tanemura H, Shimokawa K and Saji S (1998) Spread of colorectal cancer micrometastases in regional lymph nodes by reverse transcriptase-polymerase chain reactions for carcinoembryonic antigen and cytokeratin 20. *J Surg Oncol* **68**: 34–40

Gerhard M, Juhl H, Kalthoff H, Schreiber HW, Wagener C and Neumaier M (1994) Specific detection of carcinoembryonic antigen-expressing tumour cells in bone marrow aspirates by polymerase chain reaction. *J Clin Oncol* **12**: 725–729

Henke W and Loening SA (1998) Detection of illegitimate transcripts of prostate-specific antigen mRNA in blood by reverse transcription-polymerase chain reaction. *Int J Cancer* **77**: 164–165

Hermanek P (1995) pTNM and residual tumour classifications: problems of assessment and prognostic significance. *World J Surg* **19**: 184–190

Jung R, Petersen K, Krüger W, Wolf M, Wagener C, Zander A and Neumaier M (1999) Detection of micrometastasis by cytokeratin 20 RT-PCR is limited due to stable background transcription in granulocytes. *Br J Cancer* **81**: 870–873

Liefers GJ, Cleton-Janson AM, Van De Velde JH, Hermans J, Van Krieken JHJM, Cornelisse CJ and Tollenaar RAEM (1998) Micrometastases and survival in stage II colorectal cancer. *N Engl J Med* **339**: 223–228

Moll R, Loewe A, Laufer J and Franke WW (1992) Cytokeratin 20 in human carcinomas. A new histodiagnostic marker detected by monoclonal antibodies. *Am J Pathol* **140**: 427–447

Mori M, Mimori K, Inoue H, Barnard GF, Tsuji K, Nanbara S, Ueo H and Akijoshi T (1995) Detection of cancer micrometastases in lymph nodes by reverse transcriptase-polymerase chain reaction. *Cancer Res* **55**: 3417–3420

Mori M, Mimori K, Hiroaki U, Koichi T, Shiraishi T, Barnard GF, Sugimachi K and Akiyoshi T (1998) Clinical significance of molecular detection of carcinoma cells in lymph nodes and peripheral blood by reverse transcriptase-polymerase chain reaction in patients with gastrointestinal or breast carcinomas. *J Clin Oncol* **16**: 128–132

Natsugoe S, Mueller J, Stein HJ, Feith M, Hoefler H and Siewert JR (1998) Micrometastasis and tumour cell microinvolvement of lymph nodes from esophageal squamous cell carcinoma. *Cancer* **83**: 858–866

Neumaier M, Gerhard M and Wagener C (1995) Diagnosis of micrometastases by the amplification of tissue-specific genes. *Gene* **159**: 43–47

Oeberg A, Stenling R, Tavelin B and Lindmark G (1998) Are lymph node micrometastases of any clinical significance in Dukes stages A and B colorectal cancer? *Dis Colon Rectum* **41**: 1244–1249

Raj GV, Moreno JG and Gomella LG (1998) Utilization of polymerase chain reaction technology in the detection of solid tumours. *Cancer* **82**: 1419–1442

Ratto C, Sofo L, Ippoliti M, Merico M, Doglietto GB and Crucitti F (1998) Prognostic factors in colorectal cancer. *Dis Colon Rectum* **41**: 1033–1041

Ruo L and Guillem JG (1998) Surgical management of primary colorectal cancer. *Surg Oncol* **7**: 153–163

Ruud P, Fostad O and Hovig E (1999) Identification of a novel cytokeratin 19 pseudogene that may interfere with reverse transcriptase-polymerase chain reaction assays used to detect micrometastatic tumour cells. *Int J Cancer* **80**: 119–125

Schlag PM (1998) The 'sentinel node' concept: more questions raised than provided? *Oncologist* **3**: VI–VII

Schrewe H, Thopson J, Bona M, Hefta LJF, Maruya A, Hassauer M, Shively JE, Von Kleist S and Zimmermann W (1990) Cloning of the complete gene for carcinoembryonic antigen: analysis of ist promotor indicates a region conveying cell type-specific expression. *Mol Cell Biol* **10**: 2738–2748

Steele GD, Jr. (1994) The national cancer data base report on colorectal cancer. *Cancer* **74**: 1979–1989

Yun K, Merrie AEH, Gunn J, Phillips LV and McCall JL (2000) Keratin 20 is a specific marker of submicroscopic lymph node metastases in colorectal cancer: validation by K-RAS mutations. *J Pathol* **191**: 21–26

Zhang PJ, Reisner RM, Nangia R, Edge SB and Brooks JJ (1998) Effectiveness of multiple-level sectioning in detecting axillary nodal micrometastasis in breast cancer. *Arch Pathol Lab Med* **122**: 687–690

*© 2000 Cancer Research Campaign*
